# An Evolutionary Game Model for the Multi-Agent Co-Governance of Agricultural Non-Point Source Pollution Control under Intensive Management Pattern in China

**DOI:** 10.3390/ijerph17072472

**Published:** 2020-04-04

**Authors:** Lingyan Xu, Zhuoyun Zhou, Jianguo Du

**Affiliations:** 1 Management School, Jiangsu University, 301 Xuefu Road, Zhenjiang 212013, China; 2 Department of Systems Design Engineering, University of Waterloo, Waterloo, ON N2L 3G1, Canada

**Keywords:** agricultural non-point source pollution, multi-agent co-governance, evolutionary game, intensive management pattern

## Abstract

This paper focuses on the sustainable development dilemma of agricultural production in China under the pattern of intensive management, which is seriously challenged by agricultural non-point source pollution. The key to effectively break through the dilemma is to promote the co-governance of agricultural non-point source pollution control by stakeholders including local governments, new agricultural operators and traditional farmers. Accordingly, this paper discusses the interactive decision-making relationships between new agricultural operators and traditional farmers under the guidance of local governments, by constructing a trilateral evolutionary game model, as well as analyzing evolutionary cooperative stability strategies and realizing the simulation of evolution processes in different scenarios by MATLAB. The results show that new agricultural operators play a leading role in agricultural non-point source pollution control, whose strategies have effects such as technology spillover. The rewards from the superior government will support local governments in taking proactive action in the co-governance of agricultural non-point source pollution control, and then local governments can offer technical support and subsidies to new agricultural operators and traditional farmers for reducing their costs. Furthermore, this paper also finds that there are green synergy effects among the groups, where the variations of parameters and strategies by one group would affect the two others. Additionally, agricultural land operation rights transfers would cause traditional farmers to take more time to cooperate in the co-governance of agricultural non-point source pollution control. In order to promote the multi-agent co-governance of agricultural non-point source pollution control under intensive management pattern, this paper suggests that it should be necessary to reduce their costs and improve incentives, as well as to increase the common interests among groups and enhance their green synergy effects.

## 1. Introduction

The intensive management pattern in China is gradually developing under the guidance of land operation rights reformation policies, due to the fact that new agricultural operators represented by major professional households, family farms, farmer cooperatives and leading enterprises of agricultural industrialization have been emerging, and the land transferring area has accounted for 35% of the total land contracted by households since 2017 [1]. However, under the intensive management pattern, China’s agricultural production has increasingly relied on chemical fertilizers, pesticides, plastic film and other modern means, and adopted the modern agricultural production mode characterized by high input, yield and waste [2,3]. These extensive production means and modes have resulted in greatly improved efficiency of agricultural production, while sacrificed agricultural ecological environment [4,5], such as polluted rural water and soil [6,7]. In agricultural production activities, dissolved or solid pollutants, such as soil particles, nitrogen, phosphorus, and pesticide heavy metals in farmland, would cause agricultural non-point source pollution. According to China’s statistical yearbook of 2017, the average amount of nitrogen used in China reached 443 kg per hectare in 2016, far exceeding the internationally recognized safe limit of 225 kg per hectare [8]. In addition, the heavy metal spot level in farmland soil of major grain-producing areas in China has excessed to 21.5% [9]. Thus, agricultural non-point source pollution in China under the intensive management pattern has been characterized as cluster, explosion and crossover, for which the agricultural ecological environment has suffered serious damage [10,11].

In order to enhance agricultural sustainability and extend intensive production practices, China’s government has taken a series of major policies and measurements on agricultural non-point source pollution control, including adopting new technologies of agricultural non-point source pollution control (e.g., soil formula fertilization technology, constructed wetland technology, film mulching drip irrigation and zero-discharge pig-raising technologies of biological fermentation houses, etc.) and subsidies for using organic fertilizers. At the same time, the prisoner dilemma still exists in agricultural non-point source pollution control [12]. This is mainly caused by the lack of proactive motivation to pollution control among stakeholders [13,14], who are engaged in agricultural production consisting of new agricultural operators and traditional farmers [15]. Although the central government has invested and implemented a series of pollution control measures, local governments and agricultural producers have been inconsistent in the co-governance of agricultural non-point source pollution control. The reason is that local governments would deregulate agricultural non-point source pollution control and connive their polluting behavior of agricultural production to gain more local economic profits [16]. Furthermore, agricultural ecological environmental resources belong to the public goods [17], while agricultural non-point source pollution control has a typical positive externality. As such, new agricultural operators and traditional farmers’ environmental consumption behavior (e.g., discharge of pollution, improper use of modern means in agricultural production, etc.) have significant negative externalities and pollution spillover effects. These may lead to nonparticipants in agricultural non-point source pollution control gaining the same economic utility as participants. That is, rational agricultural producers will adopt the strategy of “free-riding”, and thus the dilemma of agricultural non-point source pollution control will inevitably become the tragedy of commons [18,19]. So, establishing an endogenous mechanism to encourage local governments, new agricultural operators and traditional farmers to form a multi-agent co-governance model of agricultural non-point source pollution control is the key measure to break through the current dilemma.

In fact, many researchers have explored the endogenous mechanism to break through the dilemma of agricultural non-point source pollution control by adopting the game theory which could describe stakeholders’ interactions and behavior decisions [20,21], while seldom considering stakeholders’ bounded rationality and dynamic decision processes of their mutual learning and influence. Thus, this paper is premised on the construction of a trilateral evolutionary game model of multi-agent co-governance in agricultural non-point source pollution control by stakeholders including local governments, new agricultural operators and traditional farmers. This is mainly attributed to the point that evolutionary game theory focuses on behavioral decisions among bounded rationality groups by adopting differential equations or partial differential equations, which could analyze the dynamic processes and stability of their decision-making evolutions. As many decision models are derived from evolutionary game theory, among which the most common is the replication dynamic evolutionary game model, thus the replication dynamic evolutionary game model will be adopted to explore the evolution rules of the three groups in this paper.

According to the review of relevant research on pollution control in agricultural production and rural environment based on game theory, the rest of this paper constructs the research model of the evolutionary game for the multi-agent co-governance of agricultural non-point source pollution control and analyzes the evolutionary equilibriums. Then, in Section 5, numerical simulations and scenario analysis are implemented, and accordingly, conclusions and suggestions to promote the multi-agent co-governance of agricultural non-point source pollution control under the intensive management pattern are obtained.

Thus, this paper will extend previous research by clarifying the interactive relationships between groups of local governments, new agricultural operators and traditional farmers. In particular, traditional farmers have a dual decision-making mechanism in the game; that is, traditional farmers could firstly make a decision of either to transfer the land operation rights to new agricultural operators or independently operate the land, and then decide whether to cooperate in the co-governance of agricultural non-point source pollution control. To deal with the above, this paper constructed a trilateral evolutionary game model of multi-agent co-governance in agricultural non-point source pollution control by local governments, new agricultural operators and traditional farmers. Based on the model, the cooperative stability evolution processes of these three groups’ behavior were analyzed, and, furthermore, the guiding role of local governments in the multi-agent co-governance model was also presented. Moreover, this paper put forward propositions for decision-making by analyzing the interactive behavior of these groups under different conditions and scenarios, as well as simulating the evolution processes of multi-agent co-governance in agricultural non-point source pollution control. This paper mainly contributes to the following aspects:

(1) We build an evolutionary game model to formulate multi-agent co-governance of agricultural non-point source pollution control by local governments, new agricultural operators and traditional farmers of bounded rationality.

(2) We analyze the evolutionary stable strategies and conditional requirements for how the groups could cooperate in a multi-agent co-governance model.

(3) We conduct a numerical simulation in different scenarios to propose how the multi-agent co-governance model of agricultural non-point source pollution control will eventually evolve into an asymptotically stable state.

## 2. Literature Review

Our study is closely relevant to the research on pollution control in agricultural production and rural environment based on game theory. Some of this research focused on the agents’ profits and cost allocation about pollution control. For instance, Poorsepahy et al. put forward a new game theoretical methodology to solve the problem of river pollution by adopting river pollution permit allocation in shared agricultural areas [22]. In addition, Skardi et al. used the Nash bargaining theory to fairly allocate the cost of participating players in a cooperative watershed coalition for non-point source pollution management in watersheds [20]. Other research has focused on the relationship between the government and farmers in agricultural and rural pollution control. For example, Yang proposed a game model between the government and farmers, and then put forward suggestions on developing and strengthening farmers’ cooperative organizations and integrating rural environment and farmers’ participation in the evaluation of government performance to ensure effective government supervision of rural environment [23]. More straightforwardly, Hu et al. pointed out that governmental deregulation of agricultural pollution should be the main cause by adopting game theory to analyze the regulation of grassland ecological compensation [24].

However, most of these pieces of research are based on traditional game theory that assumes players to be completely rational, while, in reality, players operate within an environment of almost bounded rationality. Therefore, researchers began to adopt the evolutionary game theory into the study of rural and agricultural environmental pollution control when considering the bounded rational players [25,26]. Zuo et al. constructed an evolutionary game model of large-scale farmers’ green farming under governmental regulation by analyzing their motivations. They found that it was difficult for large-scale farmers to realize the self-evolution of green operation mode under the complete market mechanism, while governmental regulation had a strong effect on the evolution [27]. Xu et al. analyzed the governance of rural water environments by the trilateral evolutionary game, concluding that as long as the government and enterprises take co-governance to effectively protect the interests of farmers, there would be conducive to improve rural water environments [28]. By focusing on the problem of environmental pollution of waste products in livestock enterprises, Tu and Zhang found that the main reason was the absence of proactive inspection by the local government with livestock enterprises and downstream farmers [29]. Cui et al. constructed evolutionary game models between the government and farmers, farmers and agricultural enterprises, to attempt to obtain the best stable strategy for better green technology diffusion. Their results indicated that slashing green production costs and supervision cost of government were crucial for the best stable strategy [30]. 

In summary, research on pollution control in agricultural production and rural environments by game theory should take the interactions between stakeholders and their bounded rationality into account. However, previous research has only taken local government, farmers and agricultural enterprises into the consideration of stakeholders, neglecting the relationship of comparison and learning between two types of agricultural producers as stakeholders who are new agricultural operators and traditional farmers under intensive management pattern [31]. In fact, as two groups of agricultural producers, new agricultural operators and traditional farmers will coexist for a long time in China because of the land geography and the household responsibility system [32]. Accordingly, their agricultural production behavior and strategies will directly affect the effectiveness of agricultural non-point source pollution control. As such, this paper constructed a trilateral evolutionary game model of multi-agent co-governance in agricultural non-point source pollution control by local governments, new agricultural operators and traditional farmers.

## 3. The Evolutionary Game Model for the Multi-Agent Co-Governance 

### 3.1. Problem Description and Assumptions

As a public and complex issue [33,34], agricultural non-point source pollution control is not only a responsibility of local governments, but also an obligation of relevant stakeholders engaged in agricultural production. Therefore, this paper considers that there are three groups of bounded rationality in agricultural non-point source pollution control within one district, consisting of local governments, new agricultural operators and traditional farmers. According to the institutional reform plan of the State Council in 2018 [35], many sectors in China’s governments are involved in agricultural non-point source pollution control. For example, the Ministry of Ecology and Environment in China takes the responsibility of supervising and guiding agricultural non-point source pollution control, the Ministry of Natural Resources performs duties of water resources investigation and the Ministry of Agriculture and Rural Affairs performs administrative management of farmland improvement projects on farmland and water conservancy construction. In summary, the local agencies under the jurisdiction of the above governmental sectors refer to local governments having two strategies including either actively guiding green production of other agents through supervision, punishment or incentives to achieve the goal of agricultural non-point source pollution control or not, denoted as $\left(g,\bar{g}\right)$. Accordingly, new agricultural operators also have two strategies of green production and non-green production, in which green production can be described as adopting organic fertilizer, environmental protection and pest control methods, as well as cleaning livestock, poultry manure and garbage and so on. While traditional farmers’ strategies are as follows, adopting green production in the case of independent management or supervising and denouncing new agricultural operators’ non-green production behavior in the case of transferring land operation rights, otherwise passively participating in pollution control. Thus, the strategy profile of new agricultural operators and traditional farmers can be denoted as $\left(e,\bar{e}\right)$ and $\left(h,\bar{h}\right)$, respectively.

Combined with the actual situation, some assumptions are presented as the following:

**Assumption** **1.** *The total number of each group in one district remains relatively stable, and then the group size can be normalized to 1. At the time of*t, *the proportion of local governments adopting strategy*g*among their groups is*x(t), *the proportion of new agricultural operators adopting strategy*e*and traditional farmers choosing strategy*h*among their groups is*y(t)*and*z(t), *respectively, where*0≤x(t)≤1, 0≤y(t)≤1*and*0≤z(t)≤1.

**Assumption** **2.** *The land area in this district is normalized to unit 1. Assume that the land operated by new agricultural operators derives from traditional farmers by transferring land operation rights. At the time of*t, *the ratio of land area in this district operated by new agricultural operators and traditional farmers is*α:1−α. *Thus, it can be concluded that the land area in this district operated by new agricultural operators and traditional farmers is*α*and*1−α, *respectively*.

**Assumption** **3.** *Local governments obtain their incomes*$S_{g}$*from taxes and fees, in which the special fund for agricultural non-point source pollution control is denoted as*$C_{g}$, *and there should be*$C_{g}<S_{g}$. *If local governments adopt strategy*g*and other groups adopt strategy*e*and*h, *then the local agricultural ecological environment will be improved, and accordingly, local governments can receive rewards*$\Delta S_{g}$*from the superior government. On the contrary, as long as other groups adopt strategy*$\bar{e}$*or*$\bar{h}$, *the local agricultural ecological environment will deteriorate, and thereby the performance of local governments will be negatively affected, which can be regarded as the punishment*$P_{g}$*from superior governments*.

**Assumption** **4.** *New agricultural operators obtain regular incomes*$S_{e}\alpha$*by non-green production with chemical fertilizers and pesticides, while the possible negative effect of agricultural non-point source pollution may result in future agricultural production damage on new agricultural operators and traditional farmers who have transferred land operation rights, which can be donated as*$k_{e}b_{e}\alpha$*and*$(1-k_{e})b_{e}\alpha$, *respectively, where*$0\leq k_{e}\leq 1$. *If local governments adopt strategy*g*or traditional farmers adopt strategy*h, *then new agricultural operators will be fined*$L_{e}\alpha$*by local governments and they will lose the land operation rights. On the other hand, when new agricultural operators adopt strategy*e*by using new equipment, green production technology and organic fertilizer, they should pay an additional cost*$C_{e}\alpha$. *As a result, they would obtain extra benefits*$\Delta S_{e}\alpha$*from selling green agricultural products. If local governments adopt strategy*g, *new agricultural operators would also receive incentives*$W_{e}\alpha$*from local governments*.

**Assumption** **5.** *Traditional farmers can decide to transfer land operation rights to new agricultural operators, and the income from transferring land operation rights is donated as*$D_{h}\alpha$. *In this situation, they will receive extra rewards*$\Delta W_{h}\alpha$*from local governments for supervising and denouncing pollution discharge by new agricultural operators. If traditional farmers decide to independently conduct agricultural production and use pesticides, chemical fertilizers and other non-green production modes, their regular incomes are*$S_{h}(1-\alpha )$, *while the possible pollution damage caused by agricultural non-point source pollution will also have a destructive impact on the future agricultural production and rural ecological environment, which may generate negative benefits*$(1-k_{h})b_{h}(1-\alpha )$*and*$k_{h}b_{h}(1-\alpha )$*to local governments and traditional farmers, respectively, where*$0\leq k_{h}\leq 1$. *If traditional farmers adopt organic fertilizers, environmentally friendly pest control modes, cleaning livestock and poultry manure to proactively participate in agricultural non-point source pollution control, they need to pay a certain amount of additional costs*$C_{h}(1-\alpha )$. *In this situation, they can receive the environmental protection incentives*$W_{h}(1-\alpha )$*from local governments, as well as the extra incomes*$\Delta S_{h}(1-\alpha )$*generated by selling green agricultural products.*

**Figure 1:**
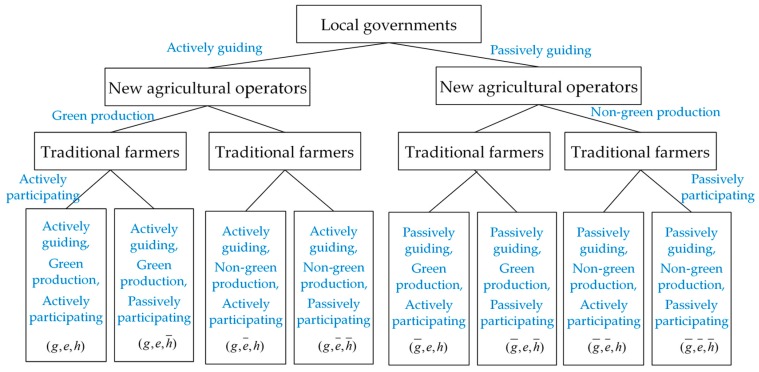
Tripartite game strategies.

According to the above assumptions, there should be a constraint of $C_{g}+\Delta S_{g}\geq W_{e}\alpha +W_{h}(1-\alpha )$, which implies that the environmental protection incentives to new agricultural operators and traditional farmers are derived from local governments’ special fund for agricultural non-point source pollution control and rewards from the superior government. Additionally, the tripartite game strategies combination of local governments, new agricultural operators and traditional farmers are shown in Figure 1.

### 3.2. Payment Matrix 

Based on the above assumptions and analysis, with different strategies, the payment matrix of this trilateral evolutionary game model of multi-agent co-governance in agricultural non-point source pollution can be conclusively shown in Table 1. As each group in this model has bounded rationality, their payoffs for each group in the matrix of Table 1 should not be less than 0.

As can be seen in Table 1, for example, the payoff of strategy (g,e,h) for each group is, respectively $S_{g}+\Delta S_{g}-C_{g}-W_{e}\alpha -(1-\alpha )W_{h}$, $(S_{e}+\Delta S_{e}-D_{h}-C_{e}+W_{e})\alpha$ and $D_{h}\alpha +(1-\alpha )(S_{h}+\Delta S_{h}-C_{h}+W_{h})$. For local governments, their payoff is the sum of $S_{g}$ from taxes and fees, and rewards $\Delta S_{g}$ from the superior government because of other groups adopting strategy e and h, then minus the special fund $C_{g}$ for agricultural non-point source pollution control and incentives $W_{e}\alpha$ and $W_{h}(1-\alpha )$ for new agricultural operators and traditional farmers to co-governance in agricultural non-point source pollution control. Similarly, their payoffs of other strategies in Table 1 can also be explained as above.

## 4. Analysis of Evolutional Stable Strategies 

### 4.1. Evolutionary Equilibrium

The expected utility of each strategy can be demonstrated based on the above analysis (Appendix A). 

Thus, the replicating dynamic equations of local governments, new agricultural operators and traditional farmers to conduct co-governance in agricultural non-point source pollution control can be composed of the following system, seen in Equation (1):

(1) $$\{\begin{array}{l} \overset{•}{x}=x(1-x)(u_{g}-u_{\bar{g}})=x(1-x)[yz\Delta S_{g}-y\alpha W_{e}-z(1-\alpha )W_{h}+(y-1)\alpha z\Delta W_{h}+(1-y)\alpha P_{e}]=x(1-x)G(x,y,z)\\ \overset{•}{y}=y(1-y)(u_{e}-u_{\bar{e}})=y(1-y)\alpha [x(W_{e}+P_{e})+zL_{{}_{e}}+\Delta S_{e}-C_{e}+k_{e}b_{e}]=y(1-y)E(x,y,z)\\ \overset{•}{z}=z(1-z)(u_{h}-u_{\bar{h}})=z(1-z)[x(1-y)\Delta W_{h}\alpha +(\Delta S_{h}-C_{h}+k_{h}b_{h})(1-\alpha )+xW_{h}(1-\alpha )]=z(1-z)H(x,y,z) \end{array}$$

Let $\overset{•}{x}=0$, $\overset{•}{y}=0$ and $\overset{•}{z}=0$, then we can derive the following lemmas about the evolutionary equilibrium.

**Lemma** **1.** *If x = 0 or 1, or y = 0 or 1, or z = 0 or 1, then*$\overset{•}{x}=0$, $\overset{•}{y}=0$*and*$\overset{•}{z}=0$. *Thus, we can derive that there are 2^3^ = 8 equilibrium solutions in this situation, which are (0,0,0), (1,0,0), (1,1,0), (1,0,1), (1,1,1), (0,1,0), (0,1,1) and (0,0,1).*

Furthermore, when x=1, 0<y<1 and 0<z<1, if $\alpha (W_{e}+P_{e}+zL_{e}+\Delta S_{e}-C_{e}+k_{e}b_{e})=0$ and $(1-y)\Delta W_{h}\alpha +(\Delta S_{h}-C_{h}+k_{h}b_{h})(1-\alpha )+W_{h}(1-\alpha )=0$, then $\overset{•}{x}=0$, $\overset{•}{y}=0$ and $\overset{•}{z}=0$. That is, if the conditions of $0<\left(C_{e}-W_{e}-P_{e}-\Delta S_{e}-k_{e}b_{e}\right)/L_{e}<1$ and $0<\left[(\Delta S_{h}-C_{h}+k_{h}b_{h})(1-\alpha )+W_{h}(1-\alpha )+\Delta W_{h}\alpha \right]/\Delta W_{h}\alpha <1$ are met, then $\left(1,[\left(\Delta S_{h}-C_{h}+k_{h}b_{h})(1-\alpha )+W_{h}(1-\alpha )+\Delta W_{h}\alpha \right]/\Delta W_{h}\alpha ,\left(C_{e}-k_{e}b_{e}-W_{e}-\Delta S_{e}-P_{e}\right)/L_{e}\right)$ is the equilibrium point of this system. Similarly, if certain conditions are met, then $\left([\left(\Delta S_{h}-C_{h}+k_{h}b_{h})(1-\alpha )\right]/\left[W_{h}(\alpha -1)-\Delta W_{h}\alpha \right],0,\alpha P_{e}/\left[(1-\alpha )W_{h}+\alpha \Delta W_{h}\right]\right)$, $\left(\left(C_{e}-k_{e}b_{e}-\Delta S_{e}\right)/\left(W_{e}+P_{e}\right),P_{e}/\left(W_{e}+P_{e}\right),0\right)$, $\left(\left(C_{h}-\Delta S_{h}-k_{h}b_{h}\right)/W_{h},1,\left(\alpha W_{e}\right)/(\Delta S_{g}-(1-\alpha )W_{h})\right)$, $\left(\left(C_{e}-k_{e}b_{e}-L_{e}-\Delta S_{e}\right)/\left(W_{e}+P_{e}\right),\left(-\alpha \Delta W_{h}+(\alpha -1)W_{h}+\alpha P_{e}\right)/\left(\alpha W_{e}-\alpha \Delta W_{h}+\alpha P_{e}-\Delta S_{g}\right),1\right)$ are all the equilibrium points of this system.

**Lemma** **2.** *When*0<x<1, 0<y<1*and*0<z<1, *if*$G(x^{*},y^{*},z^{*})=E(x^{*},y^{*},z^{*})=H(x^{*},y^{*},z^{*})=0$, *then there should be met the conditions of*$\overset{•}{x}=0$, $\overset{•}{y}=0$*and*$\overset{•}{z}=0$. *Thus, the conditions can be defined in Equation (2)*:

(2) $$\{\begin{array}{l} G(x,y,z)=[yz\Delta S_{g}-y\alpha W_{e}-z(1-\alpha )W_{h}+(y-1)\alpha z\Delta W_{h}+(1-y)\alpha P_{e}]=0\\ E(x,y,z)=\alpha [x(W_{e}+P_{e})+zL_{e}+\Delta S_{e}-C_{e}+k_{e}b_{e}]=0\\ H(x,y,z)=[x(1-y)\Delta W_{h}\alpha +(\Delta S_{h}-C_{h}+k_{h}b_{h})(1-\alpha )+xW_{h}(1-\alpha )]=0 \end{array}$$

Let $A=W_{e}+P_{e}$, $B=C_{e}-k_{e}b_{e}-\Delta S_{e}$, $C=\Delta S_{g}+\alpha \Delta W_{h}$, $D=\Delta S_{h}-C_{h}+k_{h}b_{h}$, $E=(\alpha -1)W_{h}-\alpha \Delta W_{h}$ and F={−4Eα(BC−AαLe)ΔWh[BE+AD(1−α)+αLePe]+{C[−BE+AD(1−α)]+BEαΔWh+αLe(AE+αPeΔWh)}2}12, thus two sets of solutions are obtained in Equation (3):

(3) $$\begin{array}{cc} \left\{ \begin{array}{l} x^{*}{}_{1}=\frac{\left[(\alpha -1)CAD+BCE-AE\alpha L_{e}+BE\alpha \Delta W_{h}+\alpha^{2}\Delta W_{h}L_{e}P_{e}\right]-F}{2A\Delta S_{g}E}\\ y^{*}{}_{1}=\frac{\left[(\alpha -1)CAD-BCE+AE\alpha L_{e}+BE\alpha \Delta W_{h}+\alpha^{2}\Delta W_{h}L_{e}P_{e}\right]+F}{2\alpha \Delta W_{h}(-BC+A\alpha L_{e})}\\ z^{*}{}_{1}=\frac{\left[(1-\alpha )CAD+BCE+AE\alpha L_{e}+BE\alpha \Delta W_{h}-\alpha^{2}\Delta W_{h}L_{e}P_{e}\right]+F}{2\alpha L_{e}(C+\alpha \Delta W_{h})} \end{array}\right. & \left\{ \begin{array}{l} x^{*}{}_{2}=\frac{\left[(\alpha -1)CAD+BCE-AE\alpha L_{e}+BE\alpha \Delta W_{h}+\alpha^{2}\Delta W_{h}L_{e}P_{e}\right]+F}{2A\Delta S_{g}E}\\ y^{*}{}_{2}=\frac{\left[(\alpha -1)CAD-BCE+AE\alpha L_{e}+BE\alpha \Delta W_{h}+\alpha^{2}\Delta W_{h}L_{e}P_{e}\right]-F}{2\alpha \Delta W_{h}(-BC+A\alpha L_{e})}\\ z^{*}{}_{2}=\frac{\left[(1-\alpha )CAD+BCE+AE\alpha L_{e}+BE\alpha \Delta W_{h}-\alpha^{2}\Delta W_{h}L_{e}P_{e}\right]-F}{2\alpha L_{e}(C+\alpha \Delta W_{h})} \end{array}\right. \end{array}$$

### 4.2. Analysis of Evolutional Stability Strategies and Their Conditions 

Each equilibrium point obtained above is not necessarily an evolutionary stable strategy of the system, which can be demonstrated by the local stability analysis of the Jacobian matrix. The Jacobian matrix of the system can be given in Equation (4):

(4) $$J=\left[\begin{array}{ccc} \left(1-2x)[yz\Delta S_{g}-y\alpha A-z(1-\alpha )W_{h}+(y-1)\alpha z\Delta W_{h}+\alpha P_{e}\right] & x(1-x)[z\Delta S_{g}-\alpha A+\alpha z\Delta W_{h}] & x(1-x)[y\Delta S_{g}-(1-\alpha )W_{h}+(y-1)\alpha \Delta W_{h}]\\ y(1-y)\alpha A & \left(1-2y)\alpha [xA+zL_{e}-B\right] & y(1-y)\alpha L_{e}\\ z(1-z)[(1-y)\Delta W_{h}\alpha +W_{h}(1-\alpha )] & z(1-z)(-x\Delta W_{h}\alpha ) & \left(1-2z)[x(1-y)\Delta W_{h}\alpha +D(1-\alpha )+xW_{h}(1-\alpha )\right] \end{array}\right]$$

As an equilibrium point, E_3_ (0,1,0) can be taken as an example to discuss the required conditions of the evolutionary stable strategy. Note that the Jacobian matrix of this system with an equilibrium point of E_3_ (0,1,0) is obtained in Equation (5):

(5) $$J=\left[\begin{array}{ccc} -\alpha W_{e} & 0 & 0\\ 0 & \alpha (C_{e}-\Delta S_{e}-k_{e}b_{e}) & 0\\ 0 & 0 & \left(\Delta S_{h}-C_{h}+k_{h}b_{h})(1-\alpha \right) \end{array}\right]$$

where the eigenvalues of the matrix J are $\lambda_{1}=-\alpha W_{e}$, $\lambda_{2}=\alpha (C_{e}-\Delta S_{e}-k_{e}b_{e})$, and $\lambda_{3}=(\Delta S_{h}-C_{h}+k_{h}b_{h})(1-\alpha )$. If $\lambda_{1}$, $\lambda_{2}$$\lambda_{3}$ are all less than 0, then E_3_ (0,1,0) is an evolutionary stable strategy.

Meanwhile, the Jacobian matrix of this system with an equilibrium point of E_1_ (0,0,0) is obtained in Equation (6):

(6) $$J=\left[\begin{array}{ccc} \alpha P_{e} & 0 & 0\\ 0 & \alpha (-C_{e}+k_{e}b_{e}+\Delta S_{e}) & 0\\ 0 & 0 & \left(\Delta S_{h}-C_{h}+k_{h}b_{h})(1-\alpha \right) \end{array}\right]$$

where one of the eigenvalues of the matrix J is $\lambda_{1}=\alpha P_{e}>0$, which cannot meet the required conditions of asymptotic stability. As such, E_1_ (0,0,0) is not an evolutionary stable strategy.

Similarly, other evolutionary stable strategies of the system and their required conditions can be obtained, as shown in Table 2 and Table 3.

## 5. Numerical Simulation and Scenario Analysis 

According to the replication dynamic equation and constraints of evolutionary stable strategies, numerical simulation experiments are conducted to further verify the above analysis and influence on the evolution with parameters of multi-agent’ strategies about co-governance in agricultural non-point source pollution control. Assuming that the initial time of evolution is 0 and the end time is 300, the initial proportion of local governments adopting strategy g among their groups is 0.4; meanwhile, the proportions of new agricultural operators adopting strategy e and traditional farmers’ strategy among their groups are 0.2 and 0.3, respectively. In addition, the other parameters are set as $S_{g}=0.85$*,* $S_{e}=0.7$*,* $S_{h}=0.4$*,* $P_{g}=0.2$*,* $D_{h}=0.1$*,* $C_{g}=0.1$, $P_{e}=0.2$, $W_{e}=0.1$, $L_{e}=0.25$, $C_{e}=0.3$, $k_{e}b_{e}=0.1$, $\Delta S_{e}=0.25$, $\Delta S_{h}=0.25$, $C_{h}=0.25$, $k_{h}b_{h}=0.05$, $W_{h}=0.1$, $\Delta W_{h}=0.05$, $\Delta S_{g}=0.5$ and α=0.5.

**Proposition** **1.** *In the scenario that parameters meet the required conditions of⑥, the multi-agent co-governance model of agricultural non-point source pollution control will eventually evolve into an asymptotically stable state*.

**Figure 2:**
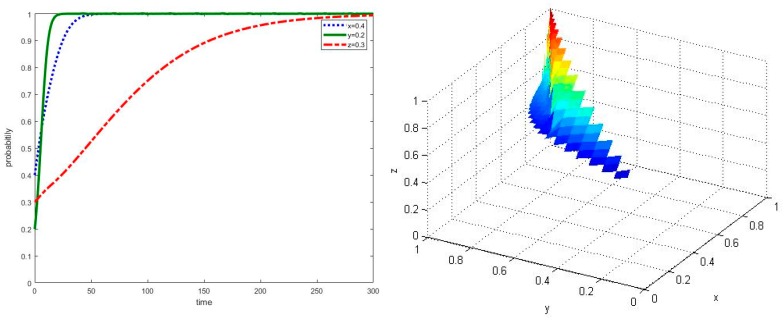
Simulation of dynamic evolution in the scenario of parameters under the conditions of ⑥.

When parameters meet the required conditions of ⑥, the simulation results of the evolution process can be shown in Figure 2. It can be seen that new agricultural operators would firstly participate in the co-governance of agricultural non-point source pollution control, followed by local governments and traditional farmers. Even if the initial proportion of new agricultural operators adopting strategy e among their groups is only 0.2, which is less than the proportion of local governments’ strategy g and traditional farmers’ strategy h among their groups, new agricultural operators are willing to cooperate in agricultural non-point source pollution control in the shortest time, which is less than t = 20. This may be related to the land operation scale of new agricultural operators, whose green production costs and extra benefits of green agricultural products will be reduced and enlarged by the influence of scale effect. Meanwhile, if new agricultural operators’ non-green production behavior is monitored and denounced by traditional farmers, new agricultural operators will lose land operation rights and be fined from local governments. Therefore, compared with other groups, new agricultural operators have stronger motivation and incentives of co-governance in agricultural non-point source pollution control.

In this scenario, an agricultural non-point source pollution control system composed of local governments, new agricultural operators and traditional farmers will eventually evolve into a collaborative state; that is, an asymptotically stable point E_8_(1,1,1) will be achieved. This shows that new agricultural operators play a leading role in the system, as such local governments should make full use of the technology spillover effect and strong motivation of new agricultural operators to guide traditional farmers participating in the multi-agent co-governance model of agricultural non-point source pollution control.

**Proposition** **2.** According to the required conditions of ⑤, the rewards provided to local governments by the superior government will greatly affect local governments’ strategies and traditional farmers’ evolutionary time of being a stable strategy.

**Figure 3:**
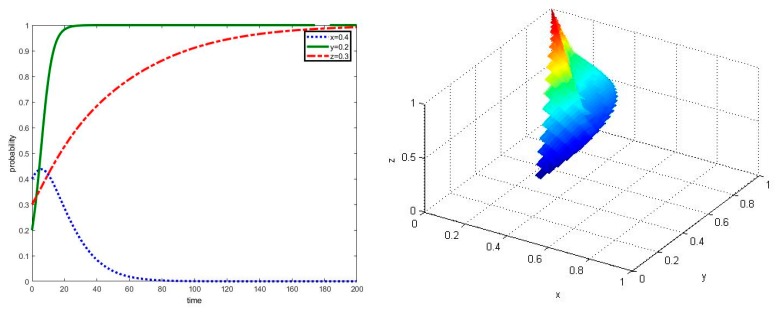
Simulation of dynamic evolution in the scenario of parameters under the conditions of ⑤.

In the scenario that parameters meet the required conditions of ⑤, we assume that the rewards provided to local governments by the superior government will be reduced to $\Delta S_{g}=0.01$, even if local governments performed well on agricultural non-point source pollution control. The simulation results can be shown in Figure 3. As seen in Figure 3, when the rewards $\Delta S_{g}$ provided to local governments by the superior government are less than the cost $\alpha W_{e}+(1-\alpha )W_{h}$ of local governments by adopting strategy g, then local governments will gradually reduce their initiatives in guiding the other two groups cooperating in the co-governance of agricultural non-point source pollution control, thus the co-governance model will no longer exist and the system will eventually tend to achieve the asymptotically stable point E_7_(0,1,1). However, in this scenario even if local governments deregulate in agricultural non-point source pollution control, new agricultural operators are still willing to cooperate in agricultural non-point source pollution control in a short time, which is near t = 20, while some traditional farmers will a take long time to cooperate in agricultural non-point source pollution control where z=1 is near t = 200.

**Proposition** **3.** According to the conditions of ③, increasing cost of green production will significantly affect the strategies of new agricultural operators and traditional farmers. The higher green production cost is, the more preference for non-green production modes they will have.

**Figure 4:**
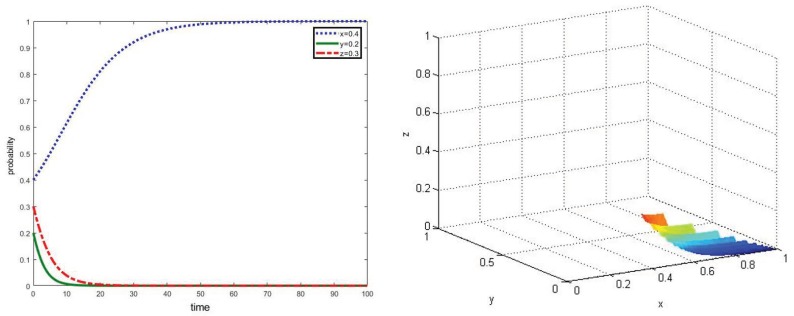
Simulation of dynamic evolution in the scenario of parameters under the conditions of ③.

In the scenario that parameters meet the required conditions of ③, the green production cost of new agricultural operators and traditional farmers are increased to $C_{e}=0.9$ and $C_{h}=0.8$ when compared with the above analysis. The simulation results can be shown in Figure 4. From the simulation results, it can be obtained that when the green production cost is higher than local governments’ subsidies, even if local governments adopt strategy g, new agricultural operators and traditional farmers are not willing to cooperate with local governments in the co-governance of agricultural non-point source pollution control. Then, the multi-agent co-governance model will collapse, and the system will eventually tend to achieve the asymptotically stable point E_4_(1,0, 0). As such, it is necessary for local governments to take measurements for providing reasonable subsidies, as well as offering technical support to reduce the green production cost of new agricultural operators and traditional farmers.

**Proposition** **4.** The proportion of land operation between new agricultural operators and traditional farmers has no significant effect on the evolution result of multi-agent co-governance in agricultural non-point source pollution control, while it has a significant influence on the evolutionary time of being a stable strategy for traditional farmers.

**Figure 5:**
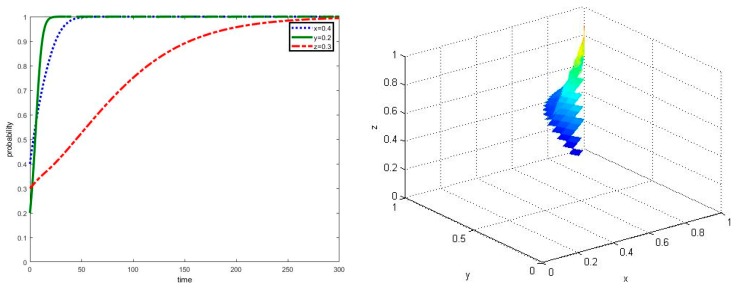
Simulation of dynamic evolution in the scenario of parameter α=0.8.

**Figure 6:**
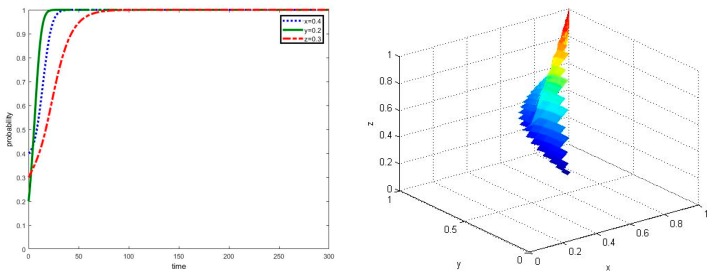
Simulation of dynamic evolution in the scenario of parameter α=0.1.

Compared to the analysis of above, the proportion of land operation by new agricultural operators has been changed, where it is set to be α=0.8 and α=0.1. In addition, the simulation results can be shown in Figure 5 and Figure 6, respectively. By the comparison of Figure 2, Figure 5 and Figure 6, it can be seen that under the certain required conditions of ⑥, the system will eventually evolve into an asymptotically stable state point E_8_ (1,1,1). While the proportion of land operation by new agricultural operators α becomes larger, traditional farmers will take more time to cooperate in the co-governance of agricultural non-point source pollution control where z=1 is near t = 300. It can be concluded that the proportion of land operation between new agricultural operators and traditional farmers has significant influence on the evolution time of being a stable strategy for traditional farmers, but it will not significantly affect the final evolution results of the system.

**Proposition** **5.** According to the required conditions of ⑦ or ⑧, there are green synergy effects among local governments, new agricultural operators and traditional farmers.

In the scenario that parameters meet the required conditions of ⑦ or ⑧, some of the local governments, new agricultural operators and traditional farmers in their groups would cooperate in the co-governance of agricultural non-point source pollution control. Assume that $W_{e}=0.1$, $P_{e}=0.2$, $L_{e}=0.25$, $C_{e}=0.3$, $k_{e}b_{e}=0.1$, $\Delta S_{e}=0.3$, $\Delta S_{h}=0.25$, $C_{h}=0.25$, $k_{h}b_{h}=0.05$, $W_{h}=0.1$, $\Delta W_{h}=0.05$, $\Delta S_{g}=0.5$, α=0.5, and let the initial proportion of local governments adopting strategy g, new agricultural operators adopting strategy e and traditional farmers’ strategy h among their groups be 0.8, 0.5 and 0.1, respectively. Then, the influence of different parameters on the dynamic evolution results of these three groups’ strategies are simulated by the tools of MATLAB. Due to the limited space, the simulation results are no longer illustrated as figures in this paper.

According to the simulation results, it can be concluded that when the rewards $\Delta S_{g}$ provided to local governments by the superior government are increased, then the initiative of local governments to take proactive actions in agricultural non-point source pollution control can be improved. Meanwhile, more and more new agricultural operators and traditional farmers would be encouraged to cooperate with local governments and actively participate in the co-governance of agricultural non-point source pollution (that is, x→1, y→1 and z→1). If the penalties $P_{e}$ for agricultural non-green production imposed by local governments on new agricultural operators are increased, new agricultural operators would be more proactive in the co-governance of agricultural non-point source pollution control, where y→1, which is also supported by Hafezalkotob [36]. Additionally, the same conclusion can be derived when the loss $L_{e}$ caused by the termination of land operation rights contract from traditional farmers to new agricultural operators, or the possible negative benefits $k_{e}b_{e}$ of agricultural non-point source pollution to new agricultural operators, or additional incomes $\Delta S_{e}$ from selling green production or incentives $W_{e}$ from local governments are raised. In particular, these parameters’ variations will also bring a certain spillover effect to the strategy g, h among local governments and traditional farmers. Similarly, if the possible negative benefits $k_{h}b_{h}$ of agricultural non-point source pollution on traditional farmers, or incentives $W_{h}$ for environmental protection from local governments, or the additional benefits $\Delta S_{h}$ of green production are increased, the initiative of traditional farmers cooperating in the co-governance of agricultural non-point source pollution control would be improved, where z→1. Besides, local governments and new agricultural operators will be actively encouraged to cooperate in the co-governance of agricultural non-point source pollution control. This conclusion is in accordance with the view proposed by Xu et al. [28] that the equilibrium point stability of one group is also affected by the other two groups’ strategy evolutions. In addition, the rewards not only provided from superior government to the local governments, but also new agricultural operators and traditional farmers obtained from local governments could be an incentive for them to cooperate in the co-governance of agricultural non-point source pollution control, which is also supported by previous research (Zuo et al. [27]; Zhang et al. [37]). 

## 6. Main Conclusions and Suggestions

### 6.1. Main Conclusions

This paper focused on the different roles, strategies and interaction among groups of local governments, new agricultural operators and traditional farmers in the co-governance of agricultural non-point source pollution control, and constructed a trilateral evolutionary game model to find the possible equilibrium points of the replication dynamic equation and the required conditions for the asymptotically stable points of the system at the equilibrium points. Numerical simulations in different scenarios have been also conducted to illustrate the effects of different parameters on the evolutionary results of multi-agent co-governance in agricultural non-point source pollution control. The results of this study indicate that the optimal strategy of this evolutionary game is (g,e,h), which corresponds to the asymptotically stable state point E_8_(1,1,1). In order to gain the optimal strategy, the superior government should provide local governments with enough rewards to take guidance so that new agricultural operators and traditional farmers would also cooperate in the co-governance of agricultural non-point source pollution control. Besides, local governments should make full use of new agricultural operators’ leading role and technology spillover effects in agricultural non-point source pollution control, as well as offer technical support and subsidies to new agricultural operators and traditional farmers so as to reduce their green production costs. Furthermore, it is also worth noting that our research shows that agricultural land operation rights transfers would cause traditional farmers to take more time to cooperate in the co-governance of agricultural non-point source pollution control, while it differs from the research of Lu and Xie [5] which found that agricultural land operation rights transfers contribute to reducing agricultural non-point source pollution.

### 6.2. Suggestions

Based on the above analysis and conclusion, this paper proposes to build a multi-agent co-governance model of agricultural non-point source pollution control by local governments, new agricultural operators and traditional farmers. Therefore, some implementations of policy design should be taken for this model. Firstly, according to the participating proportion of agents in the co-governance of agricultural non-point source pollution control mainly depending on groups’ costs and incomes, it is necessary to enhance their motivation while reducing costs in agricultural non-point source pollution control. As for local governments, the superior government should provide rewards at the beginning for agricultural non-point source pollution control through the green ecology-oriented agricultural subsidy system so as to lead them to take the first step of pollution control. Then, it is helpful for local governments to establish a dynamic guiding mechanism and enhance the consciousness of green agricultural production by comprehensive supervision and assessment on their policy effectiveness on agricultural non-point source pollution control. As for new agricultural operators and traditional farmers, it is important to reduce their green production costs by tax cuts or subsidies and increase incomes by technical innovation. Particularly, local governments should actively encourage new agricultural operators and traditional farmers to cooperate with agricultural technology companies and institutes to promote applications of green production technologies, reduce costs while improving the quality and benefits of green agricultural production. Secondly, based on the green synergy effects among these groups, the common interests among the groups should be enhanced through policy linkage and the transmission effect of market signals. Policies should not only be coordinated and linked among groups, but also should be reflected by the common desire of all groups so as to avoid the prisoner’s dilemma. Meanwhile, market mechanism of extra benefits for selling green agricultural productions can also be used to enhance the competitiveness of green agricultural products and improve the green agricultural products’ certification system, so as to increase the cognition of green agricultural products among consumers and further effectively force these three groups to cooperate in the co-governance of agricultural non-point source pollution control.

### 6.3. Limitations and Prospects

Based on the above results, there is still some important extension work to be carried out in the future, which are neglected for the sake of clarity and simplicity in this paper. Firstly, the model assumes that the total number of three groups in one district remains relatively stable, without considering the flexible and dynamic groups’ size. Secondly, the model supposes that the groups have bounded rationality, without considering their social preference. Thirdly, other stakeholders may also cooperate in the co-governance of agricultural non-point source pollution control, while the model only takes the market mechanism of extra benefits for selling green agricultural products into consideration, without discussing consumers’ preferences and purchasing decisions for green agricultural products [38], which could also affect other stakeholders’ strategies of agricultural non-point source pollution control. In addition, all of these different aspects deserve further research in the future.

## Figures and Tables

**Table 1:** The payoff matrix of the trilateral evolutionary game model.

Players				Traditional Farmers	
				h(z)	$\bar{h}(1-z)$
Local Governments	g(x)	New Agricultural Operators	e(y)	(g,e,h) $\left(\begin{array}{c} S_{g}+\Delta S_{g}-C_{g}-W_{e}\alpha -(1-\alpha )W_{h},\\ (S_{e}+\Delta S_{e}-D_{h}-C_{e}+W_{e})\alpha ,\\ D_{h}\alpha +(1-\alpha )(S_{h}+\Delta S_{h}-C_{h}+W_{h}) \end{array}\right)$	$\left(g,e,\bar{h}\right)$ $\bar{\left(\begin{array}{c} S_{g}-P_{g}-W_{e}\alpha -C_{g}-(1-k_{h})b_{h}(1-\alpha ),\\ (S_{e}+\Delta S_{e}-D_{h}-C_{e}+W_{e})\alpha ,\\ D_{h}\alpha +(S_{h}-k_{h}b_{h})(1-\alpha ) \end{array}\right)}$
			$\bar{e}(1-y)$	$\left(g,\bar{e},h\right)$ $\left(\begin{array}{c} S_{g}-P_{g}+P_{e}\alpha -W_{h}(1-\alpha )-\Delta W_{h}\alpha -C_{g},\\ (S_{e}-D_{h}-k_{e}b_{e}-L_{e}-P_{e})\alpha ,\\ \left(D_{h}-(1-k_{e})b_{e}+\Delta W_{h})\alpha +(S_{h}+\Delta S_{h}-C_{h}+W_{h})(1-\alpha \right) \end{array}\right)$	$\left(g,\bar{e},\bar{h}\right)$ $\left(\begin{array}{c} S_{g}-P_{g}+P_{e}\alpha -C_{g}-(1-k_{h})b_{h}(1-\alpha ),\\ (S_{e}-D_{h}-k_{e}b_{e}-P_{e})\alpha ,\\ \left(D_{h}-(1-k_{e})b_{e})\alpha +(S_{h}-k_{h}b_{h})(1-\alpha \right) \end{array}\right)$
	$\bar{g}(1-x)$	New Agricultural Operators	e(y)	$\left(\bar{g},e,h\right)$ $\left(\begin{array}{c} S_{g}-C_{g},\\ (S_{e}+\Delta S_{e}-D_{h}-C_{e})\alpha ,\\ D_{h}\alpha +(S_{h}+\Delta S_{h}-C_{h})(1-\alpha ) \end{array}\right)$	$\left(\bar{g},e,\bar{h}\right)$ $\left(\begin{array}{c} S_{g}-P_{g}-C_{g}-(1-k_{h})b_{h}(1-\alpha ),\\ \alpha (S_{e}+\Delta S_{e}-D_{h}-C_{e}),\\ D_{h}\alpha +(S_{h}-k_{h}b_{h})(1-\alpha ) \end{array}\right)$
			$\bar{e}(1-y)$	$\left(\bar{g},\bar{e},h\right)$ $\left(\begin{array}{c} S_{g}-P_{g}-C_{g},\\ (S_{e}-D_{h}-k_{e}b_{e}-L_{e})\alpha ,\\ \left(D_{h}-(1-k_{e})b_{e})\alpha +(S_{h}+\Delta S_{h}-C_{h})(1-\alpha \right) \end{array}\right)$	$\left(\bar{g},\bar{e},\bar{h}\right)$ $\left(\begin{array}{c} S_{g}-P_{g}-C_{g}-(1-k_{h})b_{h}(1-\alpha ),\\ (S_{e}-D_{h}-k_{e}b_{e})\alpha ,\\ \left(D_{h}-(1-k_{e})b_{e})\alpha +(S_{h}-k_{h}b_{h})(1-\alpha \right) \end{array}\right)$

**Table 2:** The evolutionary stable strategies and eigenvalues of the system.

Equilibrium Point	Eigenvalues			Asymptotically Stable
	$\lambda_{1}$	$\lambda_{2}$	$\lambda_{3}$	
E_1_(0,0,0)	$\alpha P_{e}$	$\alpha (-C_{e}+k_{e}b_{e}+\Delta S_{e})$	$\left(\Delta S_{h}-C_{h}+k_{h}b_{h})(1-\alpha \right)$	Unstable
E_2_(0,0,1)	$-(1-\alpha )W_{h}-\alpha \Delta W_{h}+\alpha P_{e}$	$\alpha (L_{e}-C_{e}+k_{e}b_{e}+\Delta S_{e})$	$-(\Delta S_{h}-C_{h}+k_{h}b_{h})(1-\alpha )$	Condition ①
E_3_(0,1,0)	$-\alpha W_{e}$	$\alpha (C_{e}-\Delta S_{e}-k_{e}b_{e})$	$\left(\Delta S_{h}-C_{h}+k_{h}b_{h})(1-\alpha \right)$	Condition ②
E_4_(1,0,0)	$-\alpha P_{e}$	$\alpha (W_{e}+P_{e}-C_{e}+k_{e}b_{e}+\Delta S_{e})$	$\Delta W_{h}\alpha +(\Delta S_{h}-C_{h}+k_{h}b_{h})(1-\alpha )+W_{h}(1-\alpha )$	Condition ③
E_5_(1,1,0)	$\alpha W_{e}$	$-\alpha (W_{e}+P_{e}-C_{e}+k_{e}b_{e}+\Delta S_{e})$	$\left(\Delta S_{h}-C_{h}+k_{h}b_{h})(1-\alpha )+W_{h}(1-\alpha \right)$	Unstable
E_6_(1,0,1)	$(1-\alpha )W_{h}+\alpha \Delta W_{h}-\alpha P_{e}$	$\alpha (W_{e}+P_{e}+L_{e}-C_{e}+k_{e}b_{e}+\Delta S_{e})$	$-[\Delta W_{h}\alpha +(\Delta S_{h}-C_{h}+k_{h}b_{h})(1-\alpha )+W_{h}(1-\alpha )]$	Condition ④
E_7_(0,1,1)	$\Delta S_{g}-\alpha W_{e}-(1-\alpha )W_{h}$	$-\alpha (L_{e}-C_{e}+k_{e}b_{e}+\Delta S_{e})$	$-(\Delta S_{h}-C_{h}+k_{h}b_{h})(1-\alpha )$	Condition ⑤
E_8_(1,1,1)	$-\Delta S_{g}+\alpha W_{e}+(1-\alpha )W_{h}$	$-\alpha (W_{e}+P_{e}+L_{e}-C_{e}+k_{e}b_{e}+\Delta S_{e})$	$-(\Delta S_{h}-C_{h}+k_{h}b_{h})(1-\alpha )-W_{h}(1-\alpha )$	Condition ⑥
E_9_$\left(1,D(1-\alpha )+W_{h}(1-\alpha )+\Delta W_{h}\alpha /\Delta W_{h}\alpha ,B-A/L_{e}\right)$	$\Delta_{1}$	$\Delta_{2}$	$-\Delta_{2}$	Unstable
E_10_$\left(D(1-\alpha )/(W_{h}(\alpha -1)-\Delta W_{h}\alpha ),0,\alpha P_{e}/((1-\alpha )W_{h}+\alpha \Delta W_{h})\right)$	$\Delta_{3}$	$\Delta_{4}$	$-\Delta_{4}$	Unstable
E_11_$\left(B/A,P_{e}/A,0\right)$	$\Delta_{5}$	$\Delta_{6}$	$-\Delta_{6}$	Unstable
E_12_$\left(-D/W_{h},1,\alpha W_{e}/\Delta S_{g}-(1-\alpha )W_{h}\right)$	$\Delta_{7}$	$\Delta_{8}$	$-\Delta_{8}$	Unstable
E_13_$\left(B-L_{e}/A,(-\alpha \Delta W_{h}+(\alpha -1)W_{h}+\alpha P_{e})/(\alpha A-\alpha \Delta W_{h}-\Delta S_{g}),1\right)$	$\Delta_{9}$	$\Delta_{10}$	$-\Delta_{10}$	Unstable
E_14_$\left(x^{*}{}_{1},y^{*}{}_{1},z^{*}{}_{1}\right)$	$\lambda_{1}{}^{*}$	$\lambda_{2}{}^{*}$	$\lambda_{3}{}^{*}$	Condition ⑦
E_15_$\left(x^{*}{}_{2},y^{*}{}_{2},z^{*}{}_{2}\right)$	$\lambda_{4}{}^{*}$	$\lambda_{5}{}^{*}$	$\lambda_{6}{}^{*}$	Condition ⑧

Note: The eigenvalues of the system $\Delta_{1}\sim \Delta_{10}$ are seen in Appendix B.

**Table 3:** The conditions of evolutionary stable strategies.

Evolutional Stable Strategies	Asymptotical Stable Conditions	Number
E_2_(0,0,1)	$-(1-\alpha )W_{h}-\alpha \Delta W_{h}+\alpha P_{e}<0,(L_{e}-C_{e}+k_{e}b_{e}+\Delta S_{e})<0,-(\Delta S_{h}-C_{h}+k_{h}b_{h})<0$	①
E_3_(0,1,0)	$-\alpha W_{e}<0,C_{e}-\Delta S_{e}-k_{e}b_{e}<0,\Delta S_{h}-C_{h}+k_{h}b_{h}<0$	②
E_4_(1,0,0)	$-\alpha P_{e}<0,W_{e}+P_{e}-C_{e}+k_{e}b_{e}+\Delta S_{e}<0,\Delta W_{h}\alpha +(\Delta S_{h}-C_{h}+k_{h}b_{h})(1-\alpha )+W_{h}(1-\alpha )<0$	③
E_6_(1,0,1)	$(1-\alpha )W_{h}+\alpha \Delta W_{h}-\alpha P_{e}<0,W_{e}+P_{e}+L_{e}-C_{e}+k_{e}b_{e}+\Delta S_{e}<0,-[\Delta W_{h}\alpha +(\Delta S_{h}-C_{h}+k_{h}b_{h})(1-\alpha )+W_{h}(1-\alpha )]<0$	④
E_7_(0,1,1) E_8_(1,1,1)	$\Delta S_{g}-\alpha W_{e}-(1-\alpha )W_{h}<0,-(L_{e}-C_{e}+k_{e}b_{e}+\Delta S_{e})<0,-(\Delta S_{h}-C_{h}+k_{h}b_{h})<0$ $-\Delta S_{g}+\alpha W_{e}+(1-\alpha )W_{h}<0,-\alpha (W_{e}+P_{e}+L_{e}-C_{e}+k_{e}b_{e}+\Delta S_{e})<0,-(\Delta S_{h}-C_{h}+k_{h}b_{h})(1-\alpha )-W_{h}(1-\alpha )<0$	⑤ ⑥
E_14_$\left(x^{*}{}_{1},y^{*}{}_{1},z^{*}{}_{1}\right)$	$\lambda_{1}{}^{*}<0,\lambda_{2}{}^{*}<0,\lambda_{3}{}^{*}<0$	⑦
E_15_$\left(x^{*}{}_{2},y^{*}{}_{2},z^{*}{}_{2}\right)$	$\lambda_{4}{}^{*}<0,\lambda_{5}{}^{*}<0,\lambda_{6}{}^{*}<0$	⑧

## Data Availability

The data used to support the findings are available in this research.

## Appendix A

The expected utility of each strategy can be demonstrated as follows:

For example, the expected utility of the strategy g by local governments is as follows:

(A1) $$\begin{array}{cc} u_{g} & =yz(S_{g}+\Delta S_{g}-C_{g}-W_{e}\alpha -(1-\alpha )W_{h})+y(1-z)(S_{g}-P_{g}-W_{e}\alpha -C_{g}-(1-k_{h})b_{h}(1-\alpha ))\\ & +(1-y)z(S_{g}-P_{g}+P_{e}\alpha -W_{h}(1-\alpha )-\Delta W_{h}\alpha -C_{g})+(1-y)(1-z)(S_{g}-P_{g}+P_{e}\alpha -C_{g}-(1-k_{h})b_{h}(1-\alpha )) \end{array}$$

While the expected utility of the strategy $\bar{g}$ by local governments is as the following:

(A2) $$\begin{array}{cc} u_{\bar{g}} & =yz(S_{g}-C_{g})+y(1-z)(S_{g}-P_{g}-C_{g}-(1-k_{h})b_{h}(1-\alpha ))+(1-y)z(S_{g}-P_{g}-C_{g})\\ & +(1-y)(1-z)(S_{g}-P_{g}-C_{g}-(1-k_{h})b_{h}(1-\alpha )) \end{array}$$

Thus, the average payoff for local governments can be described as ${\bar{u}}_{g}=xu_{g}+(1-x)u_{\bar{g}}$.

Similarly, the expected utility of the strategy e and $\bar{e}$ by new agricultural operators can be described as the following:

(A3) $$\begin{array}{cc} u_{e} & =xz\alpha (S_{e}+\Delta S_{e}-D_{h}-C_{e}+W_{e})+x(1-z)\alpha (S_{e}+\Delta S_{e}-D_{h}-C_{e}+W_{e})+(1-x)z\alpha (S_{e}+\Delta S_{e}-D_{h}-C_{e})\\ & +(1-x)(1-z)\alpha (S_{e}+\Delta S_{e}-D_{h}-C_{e}) \end{array}$$

(A4) $$\begin{array}{cc} u_{\bar{e}} & =xz\alpha (S_{e}-D_{h}-P_{e}-k_{e}b_{e}-L_{e})+x(1-z)\alpha (S_{e}-D_{h}-P_{e}-k_{e}b_{e})+(1-x)z\alpha (S_{e}-D_{h}-k_{e}b_{e}-L_{e})\\ & +(1-x)(1-z)\alpha (S_{e}-D_{h}-k_{e}b_{e}) \end{array}$$

The expected utility of the strategy h and $\bar{h}$ by traditional farmers can be described as the following:

(A5) $$\begin{array}{cc} u_{h} & =xy(D_{h}\alpha +(S_{h}+\Delta S_{h}-C_{h}+W_{h})(1-\alpha ))+x(1-y)[(D_{h}+\Delta W_{h}-(1-k_{e})b_{e})\alpha +(S_{h}+\Delta S_{h}-C_{h}+W_{h})(1-\alpha )]\\ & +(1-x)y(D_{h}\alpha +(S_{h}+\Delta S_{h}-C_{h})(1-\alpha ))+(1-x)(1-y)[(D_{h}-(1-k_{e})b_{e})\alpha +(S_{h}+\Delta S_{h}-C_{h})(1-\alpha )] \end{array}$$

(A6) $$\begin{array}{cc} u_{\bar{h}} & =xy[D_{h}\alpha +(S_{h}-k_{h}b_{h})(1-\alpha )]+x(1-y)[(D_{h}-(1-k_{e})b_{e})\alpha +(1-\alpha )(S_{h}-k_{h}b_{h})]+(1-x)y[D_{h}\alpha +(S_{h}-k_{h}b_{h})(1-\alpha )]\\ & +(1-x)(1-y)[(D_{h}-(1-k_{e})b_{e})\alpha +(1-\alpha )(S_{h}-k_{h}b_{h})] \end{array}$$

Then, the average payoff for new agricultural operators and traditional farmers can be described as ${\bar{u}}_{e}=yu_{e}+(1-y)u_{\bar{e}}$ and ${\bar{u}}_{h}=zu_{h}+(1-z)u_{\bar{h}}$.

## Appendix B

The eigenvalues of the system $\Delta_{1}\sim \Delta_{10}$ in Table 2 are as following:

$$\begin{array}{l} \Delta_{1}=\frac{-L_{e}P_{e}\alpha^{2}\Delta W_{h}+B(\Delta S_{g}((\alpha -1)W_{h}-\alpha W_{h})+D(\alpha -1)(\Delta S_{g}+\alpha \Delta W_{h}))+A(-(L_{e}\alpha +\Delta S_{g})((\alpha -1)W_{h}-\alpha \Delta W_{h})-D(\alpha -1)(L_{e}\alpha +\Delta S_{g}+\alpha \Delta W_{h}))}{L_{e}\alpha \Delta W_{h}},\\ \Delta_{2}=\frac{(A-B)\Delta W_{h}{\left(-\frac{\left(A-B-L_{e})(\alpha -1)(D+W_{h})(D-D\alpha +W_{h}-\alpha W_{h}+\alpha \Delta W_{h}\right)}{\Delta W_{h}{}^{3}}\right)}^{\left(1/2\right)}}{L_{e}},\\ \Delta_{3}=-\frac{\alpha (AD(\alpha -1)+L_{e}\alpha P_{e}+B((\alpha -1)W_{h}-\alpha \Delta W_{h}))}{(\alpha -1)W_{h}-\alpha \Delta W_{h}},\\ \Delta_{4}=\frac{{\left(-D\alpha (-\alpha +\alpha^{2})(-D+D\alpha -W_{h}+\alpha W_{h}-\alpha \Delta W_{h})(P_{e}\alpha -W_{h}+\alpha W_{h}-\alpha \Delta W_{h})\right)}^{\left(1/2\right)}}{-W_{h}+\alpha W_{h}-\alpha \Delta W_{h}},\\ \Delta_{5}=\frac{-A(\alpha -1)(AD+BW_{h})+B(A-P_{e})\alpha \Delta W_{h}}{A^{2}},\\ \Delta_{6}=\frac{\alpha {\left(B(B-A)(A-\alpha )P_{e}\right)}^{\left(1/2\right)}}{A},\\ \Delta_{7}=\frac{\alpha (W_{h}(L_{e}P_{e}\alpha +B(\Delta S_{g}+(\alpha -1)W_{h}))+A(-L_{e}P_{e}\alpha +D(\Delta S_{g}+(\alpha -1)W_{h})}{W_{h}(\Delta S_{g}+(\alpha -1)W_{h})},\\ \Delta_{8}=W_{h}(\Delta S_{g}+(\alpha -1)W_{h}){\left(\frac{DW_{e}(\alpha -1)\alpha (D+W_{h})(\Delta S_{g}-W_{h}+\alpha (-A+P_{e}+W_{h}))}{W_{h}{}^{3}{\left(\Delta S_{g}+(\alpha -1)W_{h}\right)}^{3}}\right)}^{\left(1/2\right)},\\ \Delta_{9}=\frac{A^{2}D(\alpha -1)\alpha +A((B-L_{e})\alpha ((\alpha -1)W_{h}-\alpha \Delta W_{h})-D(\alpha -1)(\Delta S_{g}+\alpha \Delta W_{h}))+(B-L_{e})(P_{e}\alpha^{2}\Delta W_{h}+\Delta S_{g}(W_{h}-\alpha W_{h}+\alpha \Delta W_{h}))}{A(A\alpha -\Delta S_{g}-\alpha \Delta W_{h})},\\ \Delta_{10}=-A(A\alpha -\Delta S_{g}-\alpha \Delta W_{h}){\left(\frac{\left(L_{e}-B)(A-B+L_{e})\alpha (A\alpha -P_{e}\alpha -\Delta S_{g}+W_{h}-W_{h}\alpha )(P_{e}\alpha -W_{h}+\alpha W_{h}-\alpha \Delta W_{h}\right)}{A^{3}(A\alpha -\Delta S_{g}-\alpha \Delta W_{h})}\right)}^{\left(1/2\right)} \end{array}$$
